# Commentary: Intranasal Oxytocin Treatment Increases Eye-Gaze Behavior toward the Owner in Ancient Japanese Dog Breeds

**DOI:** 10.3389/fpsyg.2018.01473

**Published:** 2018-08-17

**Authors:** Mattie Tops, Stephan C. J. Huijbregts, Femke T. A. Buisman-Pijlman

**Affiliations:** ^1^Department of Clinical, Neuro & Developmental Psychology, VU University Amsterdam, Amsterdam, Netherlands; ^2^Department of Clinical Child and Adolescent Studies-Neurodevelopmental Disorders, Faculty of Social Sciences, Leiden University, Leiden, Netherlands; ^3^Discipline of Pharmacology, Faculty of Health and Medical Sciences, University of Adelaide, Adelaide, SA, Australia

**Keywords:** oxytocin, habituation, familiarization, human-dog bond, stress coping, threat response, protection, social attachment

Oxytocin in mutually-gazing humans and dogs: Rewarding or stress bonding?

Nagasawa et al. (2017) build on their previous research demonstrating a positive loop between mutual eye-gazing of European dog breeds and owners and increased oxytocin during interaction (Nagasawa et al., 2015). They now investigated whether this positive loop is present during interactions between humans and ancient Japanese dog breeds. These dogs are considered to be genetically closer to wolves and more aggressive. Nagasawa's team set to increase the stressfulness of the situation for the dogs by placing two strangers in the experimentation room with the dog and its owner. Additionally, owners and strangers were instructed not to talk to each other or to talk to and touch the dog on their initiative. Oxytocin treatment of the dogs enhanced the dogs' gazing behavior and increased their owners' urinary oxytocin levels; the longer the duration of touch, the lower the increase of oxytocin in owners. Surprisingly, psychophysiological measures suggested that interaction with oxytocin-treated dogs enhanced owner's tension/arousal. Based on these and previous results, Nagasawa et al. (2015, 2017) propose the existence of an oxytocin-mediated positive loop in human-dog relationships.

Nagasawa et al. (2017) proposed that this positive loop was due to social rewarding effects of oxytocin release as seen in mother-infant relations. We feel there are other valid explanations and interpretation of these findings and want to stress the importance of considering and testing alternative explanations. As highlighted by Rault et al. (2017), it is common place in oxytocin research to assume positive valence without controlling for negative valence and arousal. Additionally, it is surprising that there is a distinction between effect of increased dog gazing and increased touch on oxytocin levels in the owner. We want to draw attention to an alternative explanation for the Nagasawa and other findings, which centers on the role of oxytocin in habituation of threat responses.

We want to draw attention to findings that demonstrated that oxytocin may alter social attention by suppressing vigilance toward threatening social stimuli, which explains many of its observed prosocial effects. Moreover, altered social attention may be implicated in a role of oxytocin in social habituation processes, particularly in mammals, whereby an individual decreases their response to an unfamiliar, dominant, or emotional social stimulus after repeated exposure (Ebitz et al., 2013; Weitekamp et al., 2017). Habituation of threat responses may be a primal function of oxytocin in mother-infant and human-dog relations (Buttner, 2016). Dogs can pose a physical threat to people and are perceived as threatening by many, for example when they are unfamiliar or angry (ÖHman, 1986). Threat appraisals in dogs are likely to be activated during gazing, because dogs tend to use eye contact as a threat among conspecifics (Bradshaw and Nott, 1995). Similarly, socially anxious humans avoid direct gaze in their social interaction, which they may perceive as threatening (Roelofs et al., 2010). Moreover, dogs look referentially to the owner after looking at an unfamiliar object and show behavioral conditioning that mirrors the owner's facial and vocal emotional message (Merola et al., 2012). This data suggests the hypothesis that dogs and humans may still show context-dependent conditioned and unconditioned responses to each-other's gaze. Such threat appraisals may trigger oxytocin responses in an attempt to habituate stress responses to unfamiliar faces and eyes (Ebitz et al., 2013). Consistent with this function, administered oxytocin decreases unconscious vigilance to threat signals from the eye region (Ebitz et al., 2013; Kanat et al., 2015). Importantly, dogs given oxytocin specifically fixated *less* on angry faces (Kis et al., 2017) and on the *eye region* of angry faces while physiological arousal was lower (Somppi et al., 2017). This highlights that oxytocin both decreased arousal and reduced eye gazing toward the eyes of angry humans, which can suggest that eye gazing was used during perceived threat.

Research from animals and human studies suggest a primal function of oxytocin in threat perception, with oxytocin reducing *aversive avoidance responses* to newborns or potential partners (Insel and Young, 2001; Cong et al., 2015). Similarly, oxytocin facilitates relaxed physiological states during reproduction-related interactions/situations that may otherwise be perceived as physically threatening, such as birth, breast feeding, and consensual sexual behavior (Porges, 2003). Interestingly, mothers displayed higher oxytocin levels after close, physical interactions with previously unknown children than with their own children (Bick and Dozier, 2010). Such findings are consistent with an association of oxytocin with coping that requires formation of trust, which in turn facilitates social stress habituation (Kéri and Kiss, 2011; Kiss et al., 2011; Tops et al., 2013a,b cf. von Dawans et al., 2012). Effective coping supported by oxytocin release, which increases social support seeking, may subsequently counter-regulate the oxytocin response itself (Tops et al., 2013b). Voluntary touch may be an important aspect of effective coping of pet dogs and their owners during perceived threat, which can than result in a counter-regulation of oxytocin (Uvnäs-Moberg et al., 2015).

Oxytocin may facilitate attachment formation between humans and dogs through its function in threat-coping responses which increase trust (Tops et al., 2013a, 2014). A primal function of oxytocin in processes of social stress habituation may also facilitate attachment between humans and dogs that are seen as guards against threats from others. In line with a role of oxytocin in protective behavior and proximity seeking to protect others, humans show increased support seeking from high-threat allies (De Dreu et al., 2012). Additionally, intranasal oxytocin induces protective aggression and in-group vs. out-group favoritism (De Dreu, 2012). Importantly, Bakermans-Kranenburg and van IJzendoorn (2017) highlight that human oxytocin studies often neglect to identify protection as an important dimension of parenting and mother-infant relations.

Studies which focus on identifying the role of oxytocin in *social reward* often neglect the potential role of *stress habituation* and do not collect measures to support or refute these distinct theories. Taking the results of Nagasawa et al. (2017) for example, it seems unlikely that the Japanese dogs after oxytocin treatment engaged in a threatening type of gazing such as staring at their owners. Still, the effects of oxytocin are believed to be context-dependent: the unfamiliar testing conditions involving two strangers and instructed inhibition of interactions were potentially stressful to both dogs and owners. The ambiguity of unnaturally suppressed interaction may have increased reassurance and protection seeking, and activation of unconditioned threat appraisals of gazing between dogs and humans.

Importantly, both threat response and positive valence may direct gazing behavior, but the current study does not let us distinguish the two. Moreover, oxytocin responses triggered by low-level threat and by social reward and protection in other contexts may still be implicated in beneficial effects of living in good relationships with dogs impacting on human well-being, coping and health (Julius et al., 2012; Kotrschal, 2016, 2018). Furthermore, oxytocin's role in stress coping responses and habituation may help explain seemingly inconsistent and context-dependent results in oxytocin research in general. For instance, several studies in humans found that intranasal oxytocin application produced different or even opposite effects depending on whether it was applied in novel or familiarized, habituated conditions (Tops, 2017). To fully understand the role of oxytocin in eye-gaze between humans and dogs, it is essential to more fully take into account valence, arousal, familiarity, and habituation (Tops et al., 2014; Rault et al., 2017).

## Author contributions

As the first author, MT was involved in all steps of the process, and was the primary writer of the text. SH and FB-P contributed to the write-up.

### Conflict of interest statement

The authors declare that the research was conducted in the absence of any commercial or financial relationships that could be construed as a potential conflict of interest.
